# Requirements for E1A dependent transcription in the yeast *Saccharomyces cerevisiae*

**DOI:** 10.1186/1471-2199-10-32

**Published:** 2009-04-17

**Authors:** Ahmed F Yousef, Christopher J Brandl, Joe S Mymryk

**Affiliations:** 1 Department of Microbiology & Immunology, University of Western Ontario, London, Ontario, Canada; 2 Department of Biochemistry, University of Western Ontario, London, Ontario, Canada; 3 Department of Oncology, University of Western Ontario, London, Ontario, Canada

## Abstract

**Background:**

The human adenovirus type 5 early region 1A (E1A) gene encodes proteins that are potent regulators of transcription. E1A does not bind DNA directly, but is recruited to target promoters by the interaction with sequence specific DNA binding proteins. In mammalian systems, E1A has been shown to contain two regions that can independently induce transcription when fused to a heterologous DNA binding domain. When expressed in *Saccharomyces cerevisiae*, each of these regions of E1A also acts as a strong transcriptional activator. This allows yeast to be used as a model system to study mechanisms by which E1A stimulates transcription.

**Results:**

Using 81 mutant yeast strains, we have evaluated the effect of deleting components of the ADA, COMPASS, CSR, INO80, ISW1, NuA3, NuA4, Mediator, PAF, RSC, SAGA, SAS, SLIK, SWI/SNF and SWR1 transcriptional regulatory complexes on E1A dependent transcription. In addition, we examined the role of histone H2B ubiquitylation by Rad6/Bre1 on transcriptional activation.

**Conclusion:**

Our analysis indicates that the two activation domains of E1A function via distinct mechanisms, identify new factors regulating E1A dependent transcription and suggest that yeast can serve as a valid model system for at least some aspects of E1A function.

## Background

**Figure 1:**
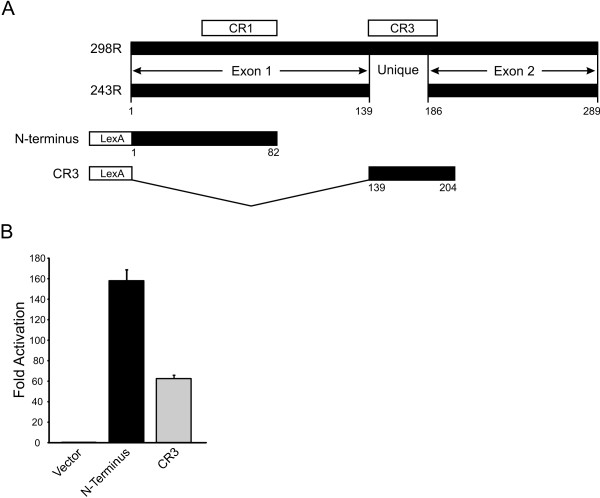
**Map of the major adenovirus type 5 E1A proteins and transcriptional activation by LexA-E1A fusions**. A) The two major products of E1A are 289 and 243 residues (R) in length and differ only by the presence of an additional 46 amino acids unique to the larger protein. Regions of sequence conservation relevant to this study (CR) are shown as are the regions expressed as LexA DBD fusions. B) Yeast strain BY4741 was transformed with the pSH1834 LexA responsive reporter plasmid and vectors expressing the LexA DBD, or the LexA DBD fused to the indicated portions of E1A. Extracts were prepared from transformed yeast and assayed for β-galactosidase activity as described previously [29]. Experiments were performed in triplicate with s.d's indicated.

Human adenovirus type 5 (HAdV-5) early region 1A (E1A) is the first viral gene expressed during infection and plays a critical role in transcriptional activation [1,2]. The primary E1A transcript is differentially spliced, yielding mRNAs encoding two major products of 289 residues (R) and 243R respectively (Figure 1A). These proteins share identical amino and carboxyl sequences and only differ by the presence of an additional 46 amino acids in the 289R protein [2,3]. The region unique to the 289R E1A protein is highly conserved amongst the E1A proteins of different adenovirus serotypes, and is referred to as conserved region 3 (CR3) [4-6]. The 289R E1A protein is thought to be primarily responsible for activation of gene expression, as mutations within CR3 generally abolish E1A transactivation [7-11]. An adjacent acidic region spanning residues 189–200, termed Auxiliary Region 1 (AR1), is also essential for efficient transactivation of early viral promoters by E1A [12].

The mechanism by which CR3 of E1A activates transcription has been studied intensely. CR3 binds numerous sequence specific transcription factors [13-17] via a promoter targeting region embedded within CR3 [15]. These interactions are thought to localize E1A to target promoters in the infected cell. When tethered to DNA by fusion to a heterologous DNA binding domain (DBD), the need for the promoter targeting region is bypassed and CR3 functions as a powerful transcriptional activator [18].

Mutations within the promoter targeting region exhibit a dominant negative effect on transcriptional activation by wild-type E1A [19,20], suggesting that these mutants sequester limiting factors necessary for transactivation by wild-type E1A. The first of these limiting factors to be identified was TBP [21]. The Sur2/TRAP150β/Med23 component of the Mediator/TRAP complex was identified to be the second critical target of CR3 [22,23]. Distinct roles for different proteasome complexes and p300/CBP in CR3 dependent transcription have also been shown [24,25].

When fused to a heterologous DBD, a second transactivation domain was identified within the N-terminal/CR1 portion of E1A [26]. This region of E1A binds multiple transcriptional regulators, including the p300, CBP (CREB Binding Protein) and pCAF acetyltransferases, TBP, TRRAP and p400 [27]. Paradoxically, this region functions as a transcriptional repression domain in the context of the E1A 243R protein by sequestering limiting factors, such as p300 and CBP, from cellular transcription factors [2]. Indeed, recent work has shown that expression of E1A 12S induces global changes in histone H3 K18 acetylation, consistent with the sequestration/retargeting of p300/CBP by E1A [28].

E1A is the product of a virus that infects human cells. However, both domains of E1A that function in mammalian cells as transcriptional activators when fused to a heterologous DBD also function as transcriptional activators in yeast [29]. Indeed, yeast have been exploited extensively as a model system to genetically study the mechanisms of E1A action [30-33,29,24].

Using a yeast model system, we have evaluated the role of histone modifying and chromatin remodelling complexes on the activity of the two transcriptional activation domains of HAdV-5 E1A. These results show that the two activation domains of E1A function via distinct but overlapping mechanisms and suggest that yeast can serve as a valid model system for identifying new targets of E1A involved in transcriptional regulation.

## Results and discussion

### LexA DBD fusions of E1A activate transcription in yeast

E1A contains two independent regions that function as transcriptional activation domains when expressed as DBD fusions in mammalian cells. We have previously shown that these same regions function as transcriptional activation domains in yeast when fused to the Gal4 DBD [29,24]. To apply yeast genetic approaches to further understand how E1A influences transcription, we assessed the role of histone modifying and chromatin remodelling complexes on the activity of these two transcriptional activation domains of HAdV-5 E1A. Specifically, we expressed the N-terminal 82 amino acids of E1A and the region spanning residues 139–204, which encompasses CR3 and AR1 of E1A, as fusions to the LexA DBD (Fig. 1A). The *E. coli *derived LexA DBD was chosen instead of the yeast Gal4 DBD to eliminate confounding effects of normal Gal4 regulation on the transcriptional activity of the E1A fusions. In addition, the LexA-E1A fusions did not inhibit yeast growth as substantially as the corresponding Gal4 DBD fusions [29]. Importantly, both portions of E1A retained transcriptional activation function as LexA DBD fusions (Fig. 1B).

### Role of the SAGA, ADA and SLIK chromatin modifying complexes

**Figure 2:**
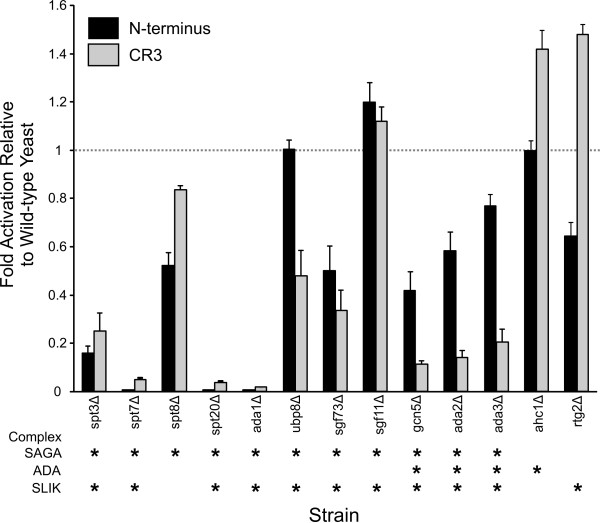
**Influence of the SAGA, ADA and SLIK complexes on E1A dependent transcriptional activation**. The indicated yeast deletion strains isogenic to BY4741 (refer to Additional file 2) were transformed with the pSH1834 LexA responsive reporter plasmid and vectors expressing the LexA DBD, or the LexA DBD fused to the indicated portions of E1A. Extracts were prepared from transformed yeast and assayed for β-galactosidase activity as described previously [29] and detailed in the Methods. Experiments were performed in triplicate with s.d's indicated. The asterisks indicate which complexes are expected to be influenced by the individual gene disruptions, as several of these genes encode factors that are components of more than one complex. For example, Spt3 is a component of the SAGA and SLIK complexes, but not the ADA complex.

We transformed plasmids expressing each E1A activation domain as a LexA DBD fusion as well as a β-galactosidase reporter gene under the control of a LexA responsive element into the wild-type yeast strain BY4741 and isogenic strains in which components of the SAGA and related ADA and SLIK complexes were disrupted (Figure 2). SAGA components can be subdivided into four general classes. Ada1, Spt7 and Spt20 are required for the structural integrity of the complex and their disruption resulted in a complete abrogation of activation dependent on the E1A N-terminus. Gcn5, Ada2 and Ada3 are necessary for acetyltransferase activity, and their disruption also impaired activation by the E1A N-terminus. Indeed, the Gcn5 acetyltransferase appeared to be the most important component of this module as its loss resulted in a 68% decrease in activity (Figure 2). Spt3 and Spt8 function in TBP recruitment by the complex and their disruption reduced activation by the N-terminus of E1A, although loss of Spt3 had a more profound effect. Recent work has shown that Spt3 directly contacts TBP and that this interaction is critical for recruiting TBP to SAGA-dependent promoters and stimulating transcription [34]. Ubp8 and Sgf11 comprise the histone deubiquitylation module in SAGA, and did not influence activation by the N-terminus of E1A. Deletion of *Sgf73 *and *Rtg2 *also reduced activation by the N-terminus of E1A, whereas the Ahc1 component of the ADA complex was not required. Based on these results, transcriptional activation by the N-terminus of E1A is primarily dependent on the components necessary to maintain the integrity of SAGA/SLIK [35] and the TBP recruitment function of Spt3 in particular, but is less dependent on the Gcn5 acetyltransferase and the Ubp8 deubiquitinase activities. Although transcriptional activation by the N-terminus of E1A in yeast does not require the ADA complex, it requires SAGA/SLIK, which may in some cases possess overlapping activities [36]. Similarly to the N-terminus, activation by E1A CR3 required the structural integrity of the SAGA complex, the TBP recruitment function of Spt3 and was independent of the ADA specific component Ahc1 (Figure 2). However, CR3 was more dependent on the SAGA acetyltransferase components (*gcn5*Δ, *ada2*Δ and *ada3*Δ) and the Ubp8 deubiquitinase, and was not influenced by the SLIK specific component Rtg2 (Figure 2). Western blot analysis confirmed that E1A was still expressed in these strains (Additional file 1).

Our previous work showed that the N-terminal and CR3 regions of E1A fused to the Gal4 DBD inhibits yeast growth in a SAGA dependent fashion. In that study, disruption of any SAGA component, including Gcn5 and Ada3/Ngg1 abrogated growth inhibition by either the N-terminus or CR3 [29]. Another study also showed that growth inhibition by the N-terminus of E1A required numerous other SAGA components [37]. Based on these observations, growth inhibition is clearly related to interaction with the SAGA complex, but is not a direct result of E1A dependent transcriptional activation. In mammalian cells, the N-terminus of E1A binds pCAF[38] and mammalian GCN5 [39], the two human orthologues of yeast Gcn5. These interactions are important, as it is known that E1A is transiently recruited to a subset of cellular promoters that are associated with cell cycle control and growth during infection. E1A induces a localized enrichment of histone acetyltransferases, including pCAF, at these loci and activates transcription [40].

### Influence of the Mediator complex on E1A dependent transcriptional activation in yeast

**Figure 3:**
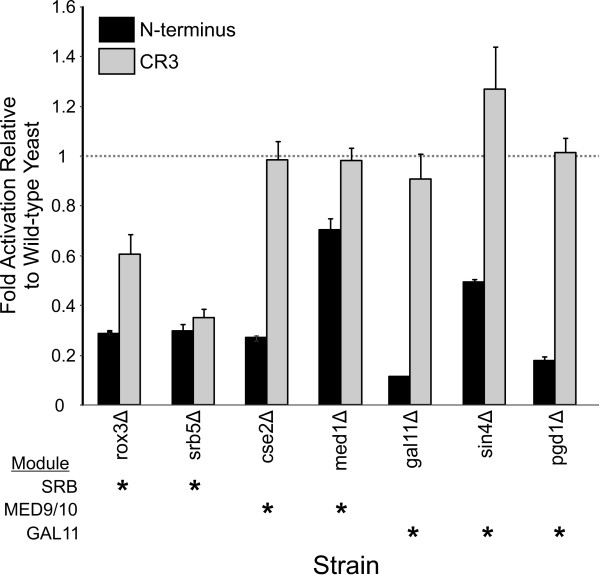
**Influence of Mediator on E1A dependent transcriptional activation in yeast**. Experiments were performed as in Figure 2. The asterisks indicate which modules are expected to be influenced by the individual gene disruptions.

In yeast, like higher eukaryotic cells, the Mediator complex interacts with RNA polymerase II and functions as both a coactivator and corepressor [41]. It is well established that E1A CR3 targets the Sur2/TRAP150β/Med23 component of the Mediator/TRAP complex in mammalian cells, which is necessary for efficient transcriptional activation [22,23]. Yeast Mediator is comprised of the Gal11, Med9/10 and Srb4 modules, and Gal11 is the Sur2/TRAP150β/Med23 orthologue [42]. We examined E1A dependent activation in strains lacking the non-essential components of the mediator complex (Figure 3). Interestingly, activation by the N-terminus of E1A decreased in all Mediator knockout strains, regardless of which module was targeted (Figure 3). This suggests that the N-terminus requires multiple Mediator modules, either directly or indirectly to stimulate transcription. In contrast, activation by CR3 was reduced only by deletion of the Srb5 or Rox3 components of the Srb4 module. Deletion of Gal11, the yeast orthologue of Sur2/TRAP150β/Med23, or the Pdg1 and Sin4 components of the Gal11 module had no effect on CR3 dependent activation. These results indicate that CR3 dependent activation in yeast is not as strongly dependent on Mediator as it is in mammalian cells.

### Role of the SWI/SNF chromatin remodelling complex in E1A dependent transcriptional activation in yeast

**Figure 4:**
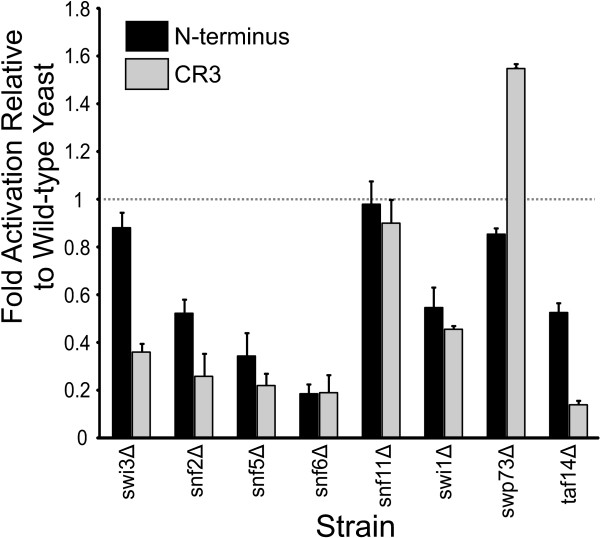
**Influence of the SWI/SNF complex on E1A dependent transcriptional activation in yeast**. Experiments were performed as in Figure 2.

The SWI/SNF complex alters chromatin structure in an ATP-dependent manner [43]. Activation by the E1A N-terminal domain fused to the LexA DBD was reduced in most yeast strains lacking components of SWI/SNF with the exception of the *swi3*Δ, *snf11*Δ and *swp73*Δ strains (Figure 4). Similarly, activation by CR3 was also decreased in yeast lacking multiple components of the SWI/SNF complex, including the *swi3*Δ strain (Figure 4). However, impairment of CR3 activation was more profound in these strains. These results suggest that the SWI/SNF dependent chromatin remodelling complex is targeted by both activation domains of E1A, but is more critical for CR3 dependent transcriptional activation. Others have reported that targeted histone acetylation by the SAGA complex predisposes promoter nucleosomes for displacement by the SWI/SNF complex, in a Snf2 dependent fashion [44]. In agreement with this, CR3 dependent activation is more dependent on the Gcn5 acetyltransferase function of SAGA and the Snf2 ATPase of the SWI/SNF complex than is activation by the N-terminus of E1A (Figures 2 and 4). A genetic screen in yeast previously identified the SWI/SNF complex as a target of the N-terminus of E1A [30]. That study demonstrated that the N-terminus of E1A blocked SWI/SNF function and inhibited yeast growth. In mammalian cells, the N-terminus of E1A interacts with p400, a SWI2 family member [45]. Although this interaction is required for oncogenic transformation by low levels of E1A [45] and suppression of EGFR expression [46], the exact effects of this interaction on transcriptional activation by E1A are not clear. A role for SWI/SNF in transcriptional activation by CR3 in mammalian cells has not been shown. Our results in yeast suggest that this could be a promising area of future investigation.

### Influence of Bre1, Rad6, COMPASS/Set1C complex and Dot1 complex on E1A dependent transcriptional activation in yeast

**Figure 5:**
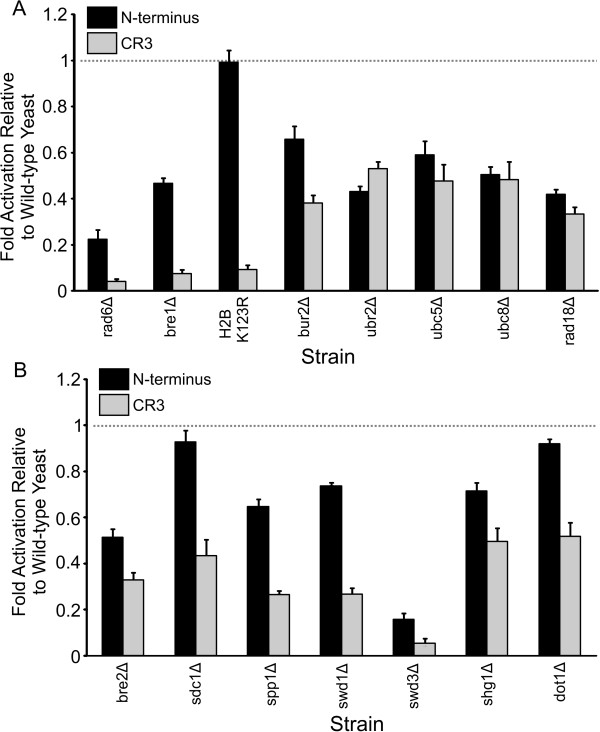
**Influence of Bre1, Rad6, COMPASS/Set1C complex and Dot1 complex on E1A dependent transcriptional activation in yeast**. Experiments were performed as in Figure 2. A) Bre1, Rad6 and related deletion strains. B) COMPASS/Set1C and Dot1 deletion strains.

Human Bre1, a histone H2B-specific ubiquitin ligase, functions as a coactivator by increasing human H2B K120 ubiquitylation and histone H3 K4 and K79 trimethylation [47,48]. CR3 activity was reduced in a *bre1*Δ strain to less than 8% activity (Figure 5A). Furthermore, disruption of *RAD6*, the corresponding ubiquitin conjugase, or conversion of the yeast H2B target lysine to arginine (K123R) also abrogated CR3 dependent activation to levels of less than 10%. Loss of Bur2, which is required for histone H2B monoubiquitination and functions as a component of the kinase complex that phosphorylates Rad6 [49], reduced CR3 dependent activation to a lesser extent. CR3 expression was not reduced in these strains (Additional file 1). However, deletion of the genes encoding the Ubc5 or Ubc8 ubiquitin conjugases, the Rad6 interacting ubiquitin ligase Ubr2, or the Rad6 interactor Rad18, which are not involved in histone ubiquitylation, reduced CR3 dependent activity to levels similar to that observed in the *bur2*Δ strain (Figure 5A). These results suggest a key role for Bre1 and Rad6 mediated ubiquitylation of H2B in CR3 dependent activation in yeast. Interestingly, deletion of the Ubp8 SAGA component, which removes the ubiquitin group from H2B K123, is also impaired for CR3 dependent activation (Figure 2). A reduction in SAGA dependent transcription has been observed previously in *ubp8*Δ yeast [50].

Similarly to CR3, transcriptional activation by the N-terminus of E1A was reduced in most of these strains (Figure 5A). However, the level of impairment was not as pronounced as observed with CR3. Unexpectedly, the reductions observed in the *bre1*Δ and *rad6*Δ strains were not reflected in a similar reduction in the H2B K123R strain, suggesting that the Bre1/Rad6 ubiquitinylation complex may have additional targets beyond H2B K123. A possibility that is supported by our observation that the Ubp8 deubiquitinase, which removes the ubiquitin moiety from H2B K123, is similarly not required for activation by the N-terminus (Figure 2).

Ubiquitylation of histone H2B K123 is required for trimethylation of histone H3 K4 and K79, modifications often associated with active transcription [51]. We next tested the role of H3 K4 methylation in E1A activity using strains lacking components of the COMPASS/Set1C complex. Deletion of Bre2, Sdc1 or Spp1, which are preferentially required to direct H3 K4 trimethylation [52], impair CR3 function (Figure 5B). Deletion of the Swd1, Swd3 and Shg1 components of the complex also reduced CR3 dependent activation (Figure 5B). CR3 expression was not reduced in these strains (Additional file 1). H3 K79 methylation in yeast is mediated by Dot1, and activation by CR3 was reduced in the *dot1*Δ strain by about 50% (Figure 5B). These results suggest that recruitment of a Bre1 orthologue and accompanying H3 K4 trimethylation may be an important component of CR3 dependent activation by E1A in mammalian cells, which deserves future study. Activation by the N-terminus of E1A was reduced to a lesser extent in these strains compared to CR3 and was not substantially affected by the deletion of Sdc1 and Dot1 (Figure 5B). These results substantiate the data presented in Figure 5A, suggesting that H2B ubiquitinylation and subsequent H3 K4 methylation are not as critical for activation by the N-terminus of E1A as they are for CR3.

### Influence of the ISW1 complexes and Spt4 on E1A dependent transcriptional activation in yeast

**Figure 6:**
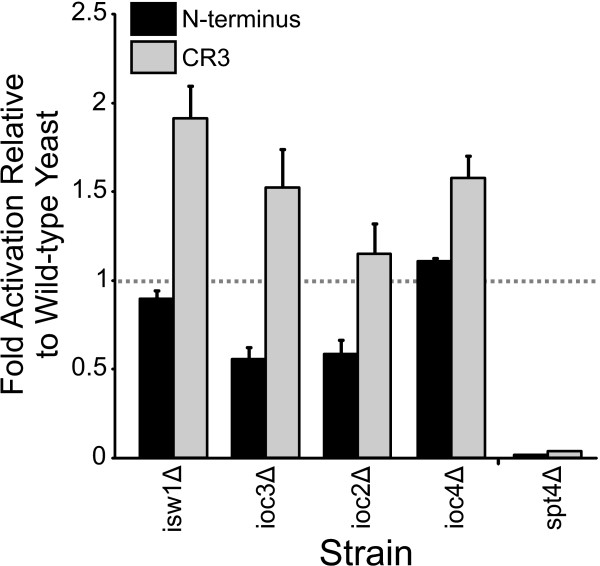
**Influence of the ISW1 complex and Spt4 on E1A dependent transcriptional activation in yeast**. Experiments were performed as in Figure 2.

H3 K4 trimethylation by Set1 has been reported to stimulate recruitment of Isw1 and its associated complexes to chromatin [53]. However, the ISW1 complexes don't play general roles in transcriptional regulation as knockout of the Isw1 ATPase component of the complex does not effect yeast growth. This complex appears to repress a subset of yeast genes as about 140 genes are activated by more than 1.5-fold in an *isw1*Δ strain [54]. Deletion of components of the ISW1 complex reduced activation by the N-terminus of E1A modestly or not at all in the case of Ioc4 (Figure 6). However, loss of any component of the complex stimulated CR3 dependent activation. Given that CR3 dependent transcriptional activation depends on COMPASS/Set1C induced H3 K4 trimethylation (Figure 5B), it would be predicted that this modification would lead to enhanced recruitment of the repressive ISW1 chromatin remodelling complexes. Thus, in the absence of ISW1 components, CR3 could function more effectively, which is what was observed.

The conversion of RNA polymerase into an elongating form is influenced by DRB Sensitivity Inducing Factor (DSIF) [55,56]. DSIF in yeast is comprised of Spt5, which is an essential protein, and Spt4 which is not. Interestingly, transcriptional activation by either portion of E1A was abrogated in the *spt4*Δ strain (Figure 6), suggesting that both activation domains of E1A also influence transcriptional elongation in yeast and that this may be a good system to further study this activity.

### Influence of the INO80, NuA3, NuA4, PAF, RSC, SAS, CSC and SWR1 complexes on E1A dependent transcriptional activation in yeast

We tested yeast strains lacking components of the INO80, RSC, SWR1 ATP dependent chromatin remodelling complexes, the NuA3, NuA4 and SAS acetylation complexes, the CSC silencing complex, the PAF lysine methyltransferase complex and several arginine methyltransferases for their effects on E1A dependent activation (Additional file 2). In general, relatively modest changes were observed, with a few exceptions where a single unique component of a complex affected E1A dependent transcription. No direct explanation could be found for these results, although we noted that many of these genes had synthetic genetic phenotypes with other transcriptional regulators essential for E1A dependent transactivation. Future developments in understanding the unique effects of these proteins may lead to additional understanding of E1A dependent transcription.

The possibility remains that disruption of certain genes could alter the copy number of the pSH1834 reporter construct used in these studies. To test this, a cassette consisting of the LexA responsive Lacz reporter was integrated into the GAL1 locus by homologous recombination in five randomly selected deletion strains. Results obtained using the integrated reporter or pSH1834 were comparable (Additional file 3), suggesting that any changes in reporter plasmid copy number caused by these individual gene disruptions did not substantially influence the results obtained in these strains. However, it remains a possibility that changes in copy number might contribute to small differences in activation in other strains.

## Conclusion

In conclusion, our analysis of the influence of chromatin remodelling and histone modifying complexes on E1A dependent activation of transcription in *S. cerevisiae *provides new evidence that there are many similarities, and some differences between transcriptional control by E1A in yeast and mammalian cells. Thus, functional analysis of E1A in yeast using genetic approaches has the potential to uncover novel mechanistic aspects of E1A function. Furthermore, genome wide analysis of E1A activity in yeast has the potential to identify novel pathways that also influence E1A function in mammalian cells.

## Methods

### Yeast strains, media, and plasmid construction

The yeast strains used along with their sources are listed in Additional file 4. Yeast culture media were prepared using standard techniques [57]. The reporter plasmid pSH1834 (8LexA operators-LacZ) was obtained from Invitrogen Corporation. A derivative of this plasmid that can be integrated into the GAL1 locus was constructed by PCR of the reporter cassette, which was subcloned into the pRS306 vector using KpnI and XbaI. The sequences of the oligos used for PCR were GCATCTAGAGGCAGCTG TCTATATGAATTACTCGAGACTAAATCTCATTCAGAAGAAGATCCCCAGCTTGGAAT and GTCGGTACCTTATTATTATTTTTGACACCAGACCAACTGG. This vector was linearized using the unique XhoI site before transforming into yeast. The N-terminus of HAdV-5 E1A (residues 1–82) and CR3 (residues 139–204) [29] were subcloned into the LexA DBD expression plasmid pBAIT [31] using EcoRI and SalI.

### E1A-mediated transcriptional activation assay: LacZ Activity

Yeast transformations were performed using a modified lithium acetate procedure [58] and plated on synthetic complete (SC) media lacking appropriate nutrients. Wild-type or mutant yeast strains were transformed with the reporter and vector or E1A expression plasmid. β-galactosidase assays were performed as previously described [29]. Briefly, colonies of transformed yeast were picked off plates with sterile wooden sticks and used to inoculate 5 ml of SC liquid media lacking appropriate nutrients. The yeast were grown to a density of A_600 _= 0.8 to 1.2. 1 ml of cultures was transferred to microcentrifuge tubes, pelleted by brief centrifugation and resuspended in 1 ml of LacZ buffer (60 mM Na_2_HPO_4_.7H_2_0, 40 mM NaH_2_PO_4_.H_2_0, 10 mM KCl, 1 mM MgSO_4_) containing 2.7 μl/ml of β-mercaptoethanol. Cells were lysed by addition of 20 μl of chloroform and 40 ul of 0.1% SDS and 1 minute of vortexing. The cell lysates were then incubated at 30°C for 15 minutes. Two hundred μl of 4 μg/ml ONPG (o-Nitrophenyl β-o-Galactopyranoside) was added to each reaction. Reactions were incubated at 30°C until the tube turned light yellow, at which point it was stopped by the addition of 500 μl of 1 M Na_2_CO_3_. The tubes were cleared by centrifuging at 21,000 g for 10 minutes. The absorbance at 420 nm was measured for each reaction. Transcriptional activity was measured in LacZ units using the formula: Activity = A420/(A600 × Volume × Time). All assays were done in triplicate. Raw data is presented in Additional file 5. Fold activation of each portion of E1A was determined on a strain by strain basis with respect to the control LexA vector. Changes in activation with respect to parental BY4741 strain yeast were calculated by dividing the mutant strain fold activation by the fold activation from BY4741 obtained within the same experiment. This was done to minimize experimental variability and tests of N-terminus and CR3 dependent activation were performed simultaneously to allow direct comparison.

### Yeast cell extracts and western blot analysis

Yeast colonies transformed with E1A expression vectors were picked from the SC selection plates and used to inoculate 5 ml of selective SC liquid media. Cultures were grown at 30°C until they reached an OD_600 _of 1.0. Cells were collected by centrifugation at 4°C (5 minutes at 1500 g). They were washed in 1 ml of ice-cold extraction buffer (10 mM Tris-HCl pH 7.5, 1× complete protease inhibitor cocktail (Roche Diagnostics). The cells were then re-suspended in 200 μl of cold extraction buffer and transferred to a 1.5 ml microtube containing 400 μl of 425–600 micron acid-washed glass beads (Sigma Aldrich). The cells were vortexed at high speed for 30 seconds and then put on ice for 30 seconds for 12 cycles. Four hundred μl of cold extraction buffer was added to the lysate which was vortexed for another 10 seconds. The tubes were then centrifuged at 21,000 g for 10 minutes at 4°C. Two hundred μl of the supernatant was transferred to clean chilled 1.5 ml microtubes. Protein concentrations of the lysates were measured using the DC Assay Kit (Bio-Rad Laboratories). Twenty μg of total protein from each sample was resolved on Novex pre-cast 5–20% gradient Tris-Glycine PAGE gel (Invitrogen). The E1A fusions were detected using rabbit polyclonal anti-LexA antibody (Millipore).

## Authors' contributions

All experimental procedures were carried out by AFY. JSM and CJB contributed to the design of the study. All authors read and approved the final manuscript.

## Supplementary Material

Additional file 1 **Western Blot analysis of LexA-DBD-CR3 expression levels in selected yeast disruption strains**. The data provided show the relative levels of expression of LexA-DBD-CR3 in selected yeast strains. Click here for file

Additional file 2 **Fold activation relative to wild-type in different yeast deletion strains isogenic to BY4741**. The data provided show the relative fold activation of LexA-DBD-N-terminus and LexA-DBD-CR3 in additional yeast disruption strains. Click here for file

Additional file 3 **Reporter copy number variation in yeast disruption strains and effect of different methods of preparing yeast extract on E1A-dependent transcriptional activation**. The data provided show the effect of changing the copy number of the reporter or method of preparing yeast extracts on E1A dependent transcriptional activation in selected yeast strains. Click here for file

Additional file 4 **List of all yeast strains and their sources that were used in this study**. This table lists all yeast strains and their sources that were used in this study. Click here for file

Additional file 5 **Raw data representing average "Miller units" obtained from performing the experiments described in the Methods**. This table lists all the raw data obtained in this study. Click here for file
